# Potential Utility of a 4K Consumer Camera for Surgical Education in Ophthalmology

**DOI:** 10.1155/2017/4374521

**Published:** 2017-05-03

**Authors:** Tsunetomo Ichihashi, Yutaka Hirabayashi, Miyuki Nagahara

**Affiliations:** Department of Ophthalmology, Tokai University Hachioji Hospital, Hachioji, Japan

## Abstract

*Purpose.* We evaluated the potential utility of a cost-effective 4K consumer video system for surgical education in ophthalmology. *Setting.* Tokai University Hachioji Hospital, Tokyo, Japan. *Design.* Experimental study. *Methods.* The eyes that underwent cataract surgery, glaucoma surgery, vitreoretinal surgery, or oculoplastic surgery between February 2016 and April 2016 were recorded with 17.2 million pixels using a high-definition digital video camera (LUMIX DMC-GH4, Panasonic, Japan) and with 0.41 million pixels using a conventional analog video camera (MKC-501, Ikegami, Japan). Motion pictures of two cases for each surgery type were evaluated and classified as having poor, normal, or excellent visibility. *Results.* The 4K video system was easily installed by reading the instructions without technical expertise. The details of the surgical picture in the 4K system were highly improved over those of the conventional pictures, and the visual effects for surgical education were significantly improved. Motion pictures were stored for approximately 11 h with 512 GB SD memory. The total price of this system was USD 8000, which is a very low price compared with a commercial system. *Conclusion.* This 4K consumer camera was able to record and play back with high-definition surgical field visibility on the 4K monitor and is a low-cost, high-performing alternative for surgical facilities.

## 1. Introduction

Surgical education in ophthalmology is performed using an observation scope in the operating room. For ideal instruction, the surgical procedure should be shared. Therefore, video teaching systems are effective tools for enhancing student competencies and technical skills [1–8]. Previously available high-definition cameras [9], however, have not been used conventionally due to their high-cost and difficult installation and operating procedures. Although the previously available conventional video systems [9–11] are not adequate to show the details of the surgical procedure because of low resolution, a high-definition video (1920 × 1080 pixels) system that can be used for practical applications recently came on the market. The visual quality of these systems, however, is low due to the lack of definition and the display. Recently, a high-definition 4K camera was developed as a consumer product available at a relatively low cost. The purpose of the present study was to evaluate the potential utility of the cost-effective 4K consumer video system for surgical education in ophthalmology.

## 2. Subject and Methods

This study adhered to the tenets of the Declaration of Helsinki and was approved by the Institutional Review Board of Tokai University Hachioji Hospital. Informed consent was obtained from the patients for the use of any videos and pictures taken while teaching the surgical procedure, including their use in medical journals. Recorded video files are not linked to the patient's medical record number.

Eyes that underwent cataract surgery, glaucoma surgery, vitreoretinal surgery, or oculoplastic surgery between February 2016 and April were recorded with a 17.2 million pixel high-definition digital video camera (LUMIX DMC-GH4, Panasonic, Japan) and a 0.41 million pixel conventional analog video camera (MKC-501, Ikegami, Japan) at the University of Tokai Hachioji Hospital. Two cases of each type of surgery were included in this study. Eyes with corneal opacity were excluded because visibility was reduced by corneal opacity, and patients under anticoagulant treatment were also excluded.

Motion pictures from each operation were evaluated and classified as having poor, normal, or excellent visibility.

## 3. Technique

**Figure 1:**
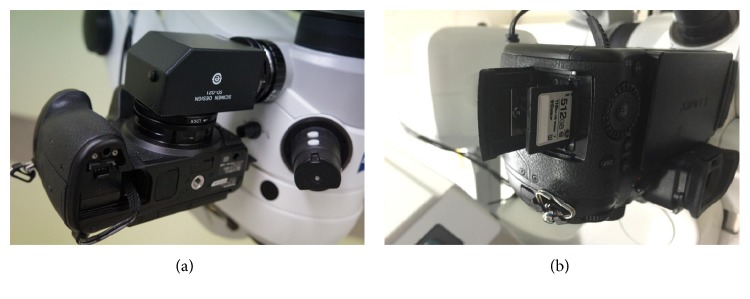
Installed image of 4K camera. (a) The LUMIX DMC-GH4 (Panasonic, Japan) was attached to the LUMELA 700 (ZEISS, Germany) surgical microscope with an SD-021 adaptor (SCIMEN Design Ltd., Japan). (b) Motion picture of 3840 × 2160 (8.29 million pixels: 4K) with 29.97 frames per second in progressive mode can be recorded on a 512 GB SD memory card for approximately 11 h.

The LUMIX body, which is a digital single-lens mirrorless consumer camera, was attached to a LUMELA 700 (ZEISS, Germany) with a mount adaptor (SD-021, Scimen Design Ltd., Japan) (Figure 1(a)). LUMIX has a Live MOS image sensor 17.3 mm × 13.0 mm in size and 17.20 million total pixels. A motion picture of 3840 × 2160 (8.29 million pixels: 4K) with 29.97 frames per second in progressive mode can be recorded on an SD memory card. The recorded file is continually divided every 4 GB (~5 min). Maximum recorded time depends on the memory volume; for example, 512 GB memory can be recorded approximately 11 h (Figure 1(b)). The real-time motion picture can be projected on a 31.5-inch 4K monitor (EV3237, EIZO, Japan) connected by an HDMI cable. A video codec, MP4 and MOV, can be selected and the video can be projected on a personal computer. Recorded files can be stored to a hard disc drive connected to a personal computer.

The conventional analog video camera, which included a 0.41 million pixel CCD camera, was attached to a LUMERA 700 with a mount adaptor (CLA-301, Ikegami, Japan). A motion picture of 740 × 480 (0.355 million pixels) with 29.97 frames per second in progressive mode can be recorded by a DVD recorder in standard mode. 4.3 GB DVD-R media were used for recording with MPEG2 video codec. Recorded file on the DVD media can be stored to the hard disc drive connected to a personal computer.

## 4. Results

**Figure 2:**
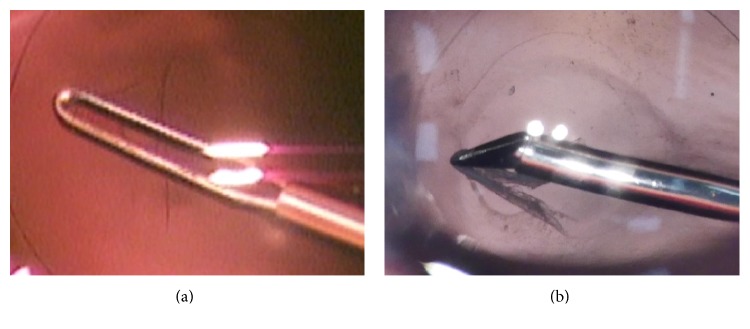
Posterior continuous curvilinear capsulorhexis (CCC) in pediatric cataract surgery. (a) The border of the posterior CCC can be partially seen in the conventional video image. (b) Almost the entire border of the posterior CCC can be seen in the 4K image.

**Figure 3:**
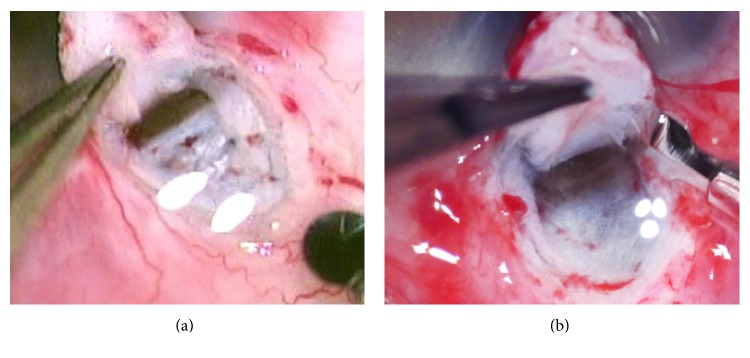
Deep sclerotomy in glaucoma surgery. (a) The border between Descemet's membrane and the juxtacanalicular tissue was detected with poor visibility in the analog video. (b) Juxtacanalicular tissue was detected with excellent visibility using the 4K camera.

**Figure 4:**
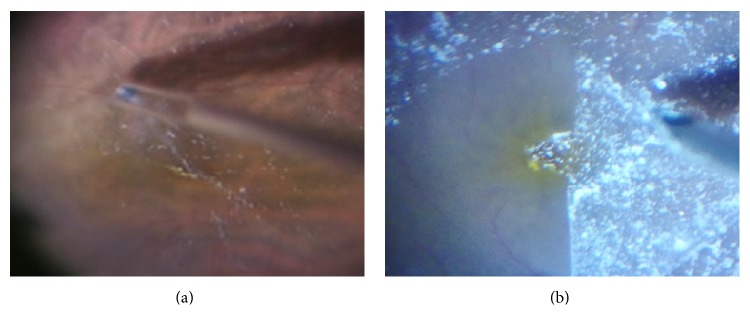
Triamcinolone acetonide-assisted internal limiting membrane (ILM) peeling in vitreoretinal surgery. (a) The border of the macular hole cannot be seen in the conventional video image. (b) The ILM with triamcinolone and the border of the macular hole can be seen in the 4K image.

**Figure 5:**
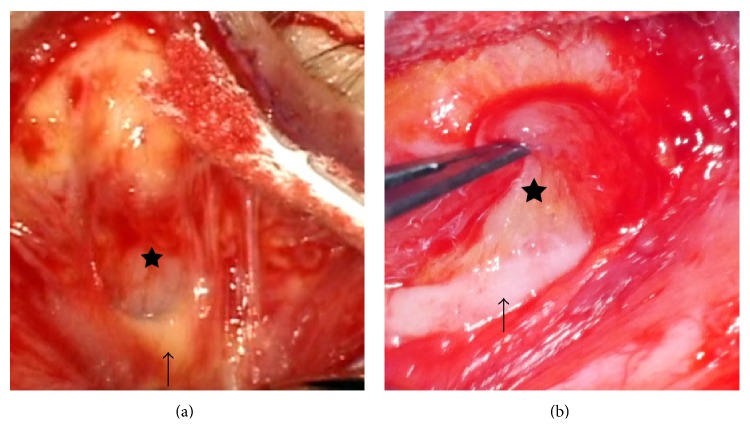
Levator resection in oculoplastic surgery. Muller's muscle (star) and Whitnall's ligament (arrow) can be seen in both images. The contrast of the image is improved in the 4K image (b) compared with that in the conventional video image (a).

This system was easily installed by reading the instructions without technical expertise. A high-definition motion picture of 3840 × 2160 with 29.97 frames per second in progressive mode was recorded on an SD memory card, and a conventional motion picture of 740 × 480 with 29.97 frames per second in progressive mode was recorded by a DVD recorder in standard mode. Motion pictures were evaluated during posterior capsulotomy in cataract surgery, deep scleral flap dissection in glaucoma, internal limiting membrane (ILM) peeling in vitreoretinal surgery, and levator resection in oculoplastic surgery. The border of the posterior capsulotomy was detected with poor visibility by analog video (Figure 2(a)), but the 4K detected it with excellent visibility. (Figure 2(b)) The border between Descemet's membrane and the juxtacanalicular tissue was detected with poor visibility in the analog video (Figure 3(a)), but the juxtacanalicular tissue was detected with excellent visibility by the 4K camera (Figure 3(b)). The ILM with triamcinolone and the border of the macular hole could not be seen in the conventional video image (Figure 4(a)), but the ILM with triamcinolone and the border of macular hole were easily visualized in the 4K image (Figure 4(b)). Although Muller's muscle and Whitnall's ligament could be seen in both images, the contrast of the image was significantly improved in the 4K image (Figure 5(b)) compared with the conventional video image (Figure 5(a)).

There is no time lag for viewing and excellent visibility on the 4K monitor, and the visibility was significantly improved compared with that of the conventional video in all cases. Details of every surgical procedure were observed on the monitor. The 4K motion pictures were stored approximately 11 h with 512 GB of SD memory. Recording files were made every 4 GB because computer operating systems such as Windows cannot use files larger than 4 GB. Pictures captured from each surgical video were stored as image files in JPEG format.

The details of the surgical procedure were recorded with MP4 codec in the 4K. The video can be played back on the computer with 4K video display capabilities, and accurate instruction can be given to enhance technical skills. The price of the 4K consumer camera is ~USD 1200 and the total price of this system is USD 8000, which is a very low price compared with a commercial system.

## 5. Discussion

A video-recording system is an effective tool for enhancing trainee competencies and technical skills, but surgical education is performed using an observation scope in the operating room, because the quality of the recording video on a conventional analog camera is not high enough to show details of the surgical procedure in ophthalmic surgery.

There are no reports of surgical recording systems using 4K consumer cameras. In human eyes, there are about six to seven million cones and are most concentrated towards the macular hole [12]. Video recording systems have been improved with electronic devices such as high-definition motion detectors, large memory systems, and high-definition monitors. The 4K displays are high quality with high-definition sensors (8.29 million pixels). We selected the LUMIX 4K camera from several consumer cameras because of its high performance, few restrictions for recording, and cost-effective production.

Recorded 4K video files can be easily played back on a personal computer. In vitreous surgery, the ILM peeling procedure was detected with good visibility. In glaucoma surgery, scleral fibers in deep scleral flap dissection were detected more clearly than those in the previous video system. In cataract surgery, details of the anterior segment during surgery were detected with excellent visibility. In levator resection, it was easy to discern the levator aponeurosis and Muller's muscle on the monitor.

The 4K consumer camera has made rapid progress in these days. The price of the 4K consumer camera is ~USD 1200, and the total price of this system is USD 8000, which is a very low price compared with that of a commercial system. Specifications of 4K camera are often improving or developing new kinds of products. Advantage of use of a consumer camera is renewable by inexpensiveness.

Therefore, this 4K system allowed for ideal instruction with playback video files after surgery and visual effects significantly improved surgical education.

## 6. Conclusion

We described the potential utility of the 4K consumer camera for surgical education in ophthalmology. Although there are many available options for recording surgical fields, this system is able to both record and play back with a high-definition surgical field visibility on the 4K monitor. Each device has distinctive characteristics, advantages, and disadvantages. Recording devices that best meet the needs of the surgical situation should be selected, but the consumer camera is an effective low-cost option for surgical facilities.
